# Recombinant expression, purification and biochemical characterization of kievitone hydratase from *Nectria haematococca*

**DOI:** 10.1371/journal.pone.0192653

**Published:** 2018-02-08

**Authors:** Matthias Engleder, Melissa Horvat, Anita Emmerstorfer-Augustin, Tamara Wriessnegger, Stefanie Gabriel, Gernot Strohmeier, Hansjörg Weber, Monika Müller, Iwona Kaluzna, Daniel Mink, Martin Schürmann, Harald Pichler

**Affiliations:** 1 acib—Austrian Centre of Industrial Biotechnology, Graz, Austria; 2 Institute of Molecular Biotechnology, Graz University of Technology, NAWI Graz, BioTechMed Graz, Graz, Austria; 3 Institute of Organic Chemistry, Graz University of Technology, NAWI Graz, Graz, Austria; 4 DSM Ahead R&D—Innovative Synthesis, Geleen, The Netherlands; Weizmann Institute of Science, ISRAEL

## Abstract

Kievitone hydratase catalyzes the addition of water to the double bond of the prenyl moiety of plant isoflavonoid kievitone and, thereby, forms the tertiary alcohol hydroxy-kievitone. In nature, this conversion is associated with a defense mechanism of fungal pathogens against phytoalexins generated by host plants after infection. As of today, a gene sequence coding for kievitone hydratase activity has only been identified and characterized in *Fusarium solani* f. sp. *phaseoli*. Here, we report on the identification of a putative kievitone hydratase sequence in *Nectria haematococca* (*Nh*KHS), the teleomorph state of *F*. *solani*, based on *in silico* sequence analyses. After heterologous expression of the enzyme in the methylotrophic yeast *Pichia pastoris*, we have confirmed its kievitone hydration activity and have assessed its biochemical properties and substrate specificity. Purified recombinant *Nh*KHS is obviously a homodimeric glycoprotein. Due to its good activity for the readily available chalcone derivative xanthohumol (XN), this compound was selected as a model substrate for biochemical studies. The optimal pH and temperature for hydratase activity were 6.0 and 35°C, respectively, and apparent V_max_ and K_m_ values for hydration of XN were 7.16 μmol min^-1^ mg^-1^ and 0.98 ± 0.13 mM, respectively. Due to its catalytic properties and apparent substrate promiscuity, *Nh*KHS is a promising enzyme for the biocatalytic production of tertiary alcohols.

## Introduction

The production of enantiopure tertiary alcohols is a major challenge of organic synthesis as these functional groups are widely applicable for the generation of pharmaceuticals or other bioactive compounds [1,2]. However, synthesis of tertiary alcohols is still a demanding task for synthetic organic chemistry due to issues such as low yields, poor selectivity or harsh reaction conditions. Therefore, sustainable biocatalytic processes that rely on enzymatic transformations are highly desirable [2,3]. The most extensively applied enzymes for synthesis of optically active, tertiary alcohols belong to the class of hydrolases [4,5], where especially lipases and esterases have been used for their kinetic resolution [1,2]. In contrast, members of other enzyme classes are markedly underrepresented, even given the fact that some of them would offer great possibilities for applications. This holds particularly true for hydro-lyases (EC 4.2.1.X), which are able to catalyze the highly selective, reversible addition of water to non-activated carbon-carbon double bonds and, thereby, generate primary, secondary or tertiary alcohols [6,7]. Aside from cofactor dependent hydro-lyases, a number of enzymes catalyze the addition of water cofactor-independently, which increases the potential of this enzyme group for industrial applications. However, although more than 100 hydro-lyases have been discovered to date, only a very limited number has been applied industrially. The most prominent examples include nitrile hydratase and fumarase for the production of acrylamide on a 30,000 t a^-1^ scale, or the production of (S)-malic acid (2,500 t a^-1^), respectively [7]. Another enzyme group that has recently received increasing attention of both academic and industrial research are oleate hydratases. Representatives of this class of hydratases have been applied for the production of 10-hydroxystearic acid from oleic acid, as well as α,ω-dicarboxylic acids, ω-hydroxycarboxylic acids and γ-dodecalactones in multistep enzymatic reaction systems [8–10]. Compounds obtained from oleate hydratase reactions are applied widespread in the production of a large variety of chemicals and intermediates, such as polymers, coatings, lubricants, personal care, perfumes and food additives [11].

An attractive member of the hydro-lyase enzyme group for the biosynthesis of tertiary alcohols is kievitone hydratase (KHS; EC 4.2.1.95). This enzyme was first described by Kuhn and Smith in 1979 by analysis of liquid cultures of the fungal plant pathogen *Fusarium solani* f. sp. *phaseoli* [12]. It catalyzes the detoxification of the plant phytoalexin kievitone (KV) produced by *Phaseolus vulgaris* (French bean) after a microbial infection. Hydration of the isolated carbon-carbon double bond of KV results in formation of the less toxic tertiary alcohol hydroxy–kievitone (HO-KV). Thereby, KHS plays an important role in the pathogenicity of the fungus for its plant host. The first investigations of *F*. *solani* f. sp. *phaseoli* KHS (*Fs*KHS) were performed by Cleveland and Smith upon partial purification from cell free culture filtrates [13]. *Fs*KHS is an extracellular glycoprotein with inferred activity not only for the plant isoflavanon KV, but also for the pterocarpan phaseollidin. However, additional studies showed that in *F*. *solani* f. sp. *phaseoli*, kievitone hydratase and phaseollidin hydratase activities are most likely conferred by two different enzymes [14]. In 1995, the complete nucleotide sequence of *Fs*KHS was published [15]. It could be shown that secretion of the enzyme is most likely mediated by an *N*-terminal signal peptide that was not present in the mature enzyme. Using low-stringency Southern blot hybridization, several sequences homologous to *Fs*KHS were identified in different members of the *F*. *solani* species complex. This included, among others, *Nectria haematococca*, the teleomorphic state of *F*. *solani*. This corroborated an earlier study that detected HO-KV in cell free culture filtrates of *N*. *haematococca* upon infecting *P*. *vulgaris* [16]. However, to date no gene or protein sequence could be unambiguously assigned to kievitone hydratase activity in *N*. *haematococca*.

In order to elucidate potential hydro-lyases for the synthesis of tertiary alcohols, we have investigated different gene sequences from the *F*. *solani* species complex on the basis of information from previous studies [14–16]. After identifying a putative kievitone hydratase sequence in *N*. *haematococca* MP VI, we have heterologously expressed the gene in *P*. *pastoris* and confirmed its activity for KV and other flavonoids. Subsequently, a biochemical characterization provided detailed insight into the potential of this enzyme group for biocatalytic applications.

## Materials and methods

### Chemicals and media components

Standard laboratory reagents were obtained from Sigma-Aldrich (Vienna, Austria) or Carl Roth GmbH & Co. KG (Karlsruhe, Germany) with the highest purity available. KV and HO-KV were obtained from InnoSyn B.V. (Geleen, The Netherlands). 8-Prenylnaringenin was purchased from Sigma-Aldrich (Vienna, Austria), and Isoxanthohumol was a kind gift of Prof. Michael Murkovic, Institute of Biochemistry, Graz University of Technology. Hop extract capsules were purchased from Allcura (Wertheim, Germany). Restriction enzymes were acquired from Thermo Scientific (St. Leon-Rot, Germany). Bacto™ peptone, Bacto™ yeast extract and Difco™ yeast nitrogen base w/o amino acids (YNB) were obtained from Becton, Dickinson and Company (Schwechat, Austria). Zeocin^TM^ was purchased from InvivoGen (Vienna, Austria). Sterile water was acquired from Fresenius Kabi, Graz, Austria.

*P*. *pastoris* cultures were routinely grown in buffered glycerol-complex medium BMGY (1% yeast extract, 2% peptone, 100 mM potassium phosphate, pH 6.0, 1.34% YNB, 4 × 10^−5^% biotin, 1% glycerol). Buffered methanol-complex medium, BMMY (1% yeast extract, 2% peptone, 100 mM potassium phosphate, pH 6.0, 1.34% YNB, 4 × 10^−5^% biotin, 1% methanol) was used as induction medium.

### Vector and *P*. *pastoris* KHS strain construction

The *P*. *pastoris* (*Komagataella phaffii*) strain CBS7435 (NRRL Y-11430) (Sturmberger et al. 2016, Küberl et al. 2011) was used as wild type host strain for expression of kievitone hydratases from *N*. *haematococca* (*Nh*KHS; GenBank number: EEU35471.1) and *F*. *solani* (*Fs*KHS; GenBank number: L39639.1). Codon-harmonized gene variants of KHSs were designed manually by applying *the P*. *pastoris* codon usage. The synthetic KHS genes with a C-terminal His_10_-tag and *EcoR*I/*Not*I restriction sites for cloning into *P*. *pastoris* expression vectors were purchased from GeneArt®. The *P*. *pastoris* vectors p*Pp*T4_S and p*Pp*T4_Alpha_S [17] were used for intracellular or secrectory protein production. Primers used for cloning of the KHS genes from *N*. *haematococca* and *F*. *solani* into the respective vectors are given in (S1 Table). The correct nucleotide sequence of each construct was checked by sequencing the expression cassette. Expression vectors were linearized with *Smi*I for integration into the genome of *P*. *pastoris* generating the expression strain *PpKHS*Alpha. Routinely, electrocompetent *P*. *pastoris* cells were transformed with 3 μg of linearized plasmids according to the protocol of Lin-Cereghino [18]. Aliquots of transformed cells were plated on YPD containing 100 mg L^-1^ Zeocin^TM^.

### *In silico* analysis of KHS sequences

DNA and protein sequences similar to *Fs*KHS(15) were identified with the Basic Local Alignment Search Tool (BLAST) [19] using default settings. In order to find putative KHS sequences in different *N*. *haematococca* MPs, the pBLAST algorithm with *N*. *haematococca* MPs I, IV, V, and VI as search set was applied. BLASTn and tBLASTn options were selected with the nucleotide collection (nr/nt). A multiple sequence alignment of selected proteins was performed with the Clustal Omega sequence alignment tool. The amino acid sequences of (putative) KHS enzymes from *F*. *solani* (AAA87627.1), *N*. *haematococca*. (XP_003041184), *Aspergillus terreus* (XP_001217367) and *Aspergillus nidulans* (XP_682503) were compared. In addition, the putative KHS sequence from *N*. *haematococca* was subjected to analysis with SignalP to predict the presence and location of a supposed signal peptide and its cleavage site, respectively.

### Shake flask cultivations

*P*. *pastoris* main cultures were grown in 25 mL of BMGY, inoculated to a final OD_600_ of 0.1 with a pre-culture grown for 48 h in BMGY, and the cells were cultivated at 130 rpm and 28°C for 24 h. Then, 25 mL of BMMY with 1% of methanol were added yielding a final concentration of 0.5% of methanol in the culture medium. Methanol was added every 12 h to a final concentration of 0.5% for 48 h of induction. For cultivation of cells, 300 mL wide necked, baffled shake flasks covered with two layers of cotton cloth were used. For purification of recombinantly expressed His_10_-tagged *Nh*KHS, cultures were scaled-up to 400 mL in 2 L baffled shake flasks. Cells were harvested by centrifugation after 48 h of protein expression. Recombinant *Nh*KHS recovered from the culture supernatant was used in all further analyses.

### Purification of recombinant protein from *P*. *pastoris* culture supernatant

Prior to purification, the culture supernatant containing secreted *Nh*KHS was filtered through 0.22 μm filters (Millipore, Bedford, MA). His_10_-tagged *Nh*KHS was purified with Ni-NTA affinity chromatography using self-packed columns (GE Healthcare, UK). Ni-sepharose beads were prepared and regenerated according to the GE Healthcare manual. After equilibration with 50 mM NaH_2_PO_4_, pH 7.0, containing 300 mM NaCl and 10 mM imidazole, the filtered culture supernatant was loaded. Afterwards, the column was washed twice with 5 column volumes (CV) of the same buffer containing 10 mM imidazole in the first and 50 mM imidazole in the second step, respectively. Recombinant *Nh*KHS was eluted with 3 CV of 50 mM NaH_2_PO_4_, pH 7.0, containing 300 mM NaCl and 250 mM imidazole. The eluate was collected and, immediately, buffer was exchanged with 50 mM sodium citrate, pH 6.0, using disposable PD-10 desalting columns (GE Healthcare, UK) according to the manufacturer’s recommendation.

The apparent relative molecular mass of the native enzyme was determined by gel filtration on a Superdex 200 HiLoad 16/60 column (GE Healthcare, UK) in 50 mM sodium citrate, pH 6.0, and comparison of the size of the eluted protein with standard proteins. A calibration curve was generated with the standard proteins conalbumin (75 kDa), ovalbumin (44 kDa), carbonic anhydrase (29 kDa), ribonuclease A (13.7 kDa) and aprotinin (6.5 kDa).

### Fed-batch cultivation in 2 L bioreactor

For up-scaling of *Nh*KHS protein production in *P*. *pastoris*, a DASGIP parallel bioreactor system was used (DS1500TPSS; DASGIP AG, Jülich, Germany). The bioreactor cultivation was performed according to the protocol described by Wriessnegger et al. [20], applying a single instead of a two-phase cultivation process. *Pp*CBS7435 wild type and *PpKHS*Alpha strains were cultivated in parallel and the methanol induction was carried out for 123 h with a flow rate of 5 mL h^-1^. Biomass concentration in the cultivation broth was determined gravimetrically as cell dry weight (CDW). Every 24 h, 1 mL samples of the cell culture were transferred to pre-weighed 1.5 mL tubes and centrifuged for 5 min at 16,000 x g. The pellets were dried at 100°C in an oven for at least 48 h to constant weight. The supernatants were transferred into a new tube and were used for subsequent analysis of protein concentration, protein expression levels by SDS-PAGE and *Nh*KHS activity.

### Analysis of recombinant protein in the *P*. *pastoris* culture supernatant

Proteins from culture supernatant were precipitated using the chloroform/methanol method. Therefore, 400 μL of cell broth were harvested by centrifugation and the supernatant was transferred into 2.0 mL reaction tubes. A mixture of 480 μL of methanol, 160 μL of CHCl_3_ and 640 μL of ddH_2_O were added to the culture supernatants and briefly mixed. After centrifugation at full speed in a table top centrifuge for 5 min, the precipitated protein was found at the interphase. The upper aqueous layer was carefully removed and 300 μL of methanol were added to the organic phase. After centrifugation at full speed for 30 min at 4°C, the supernatant was removed and the pellet was dried at 65°C for 5 min. The dried pellet was dissolved in 30 μL of NuPAGE® loading dye at 70°C for 10 min and loaded onto a NuPAGE® SDS-gel. SDS-PAGE was performed according to the manual of the NuPAGE® SDS-PAGE System (life technologies, Vienna, Austria).

### UV-Vis spectroscopy

UV-Vis absorption spectra of His_10_-tag purified KHS were recorded from 250 nm to 1,000 nm with a Specord 205 double-beam spectrophotometer (Analytik Jena, Germany) in semi-micro quartz cuvettes with a path length of 1 cm. Spectral measurements were performed in 50 mM sodium citrate, pH 6.0. The concentration of purified protein was estimated with an ε_280_ of 83,310 M^-1^ cm^-1^.

### Determination of KHS activity

The activity of the *Nh*KHS protein was determined by applying *in vitro* assays with either culture supernatant containing secreted *Nh*KHS or His_10_-tag purified *Nh*KHS. For this task, 98 μL of culture supernatant (~ 0.6 mg mL^-1^ of *Nh*KHS) were incubated with 2 mM of substrate (KV, xanthohumol (XN), isoxanthohumol or 8-prenylnaringenin) in Pyrex reaction tubes shaken for 3 h at 150 rpm and 35°C. The reaction was stopped by adding 300 μL of methanol and formic acid to 1% final concentration. After centrifugation at 13,200 rpm for 5 min, the assay reaction was analyzed by HPLC-MS. In standard *in vitro* assays with purified *Nh*KHS, One hundred μg of protein were incubated with 0.5 mM of XN in 100 μl of 50 mM sodium citrate, pH 6.0. The reaction was incubated for 10 min at 150 rpm and 35°C, and the protein was precipitated with methanol as described.

### Enzyme kinetics

Kinetic parameters of *Nh*KHS for conversion of XN were determined using 0.05 mg mL^-1^ of purified enzyme. Substrate concentrations ranging from 0.125 mM to 3.0 mM were applied. The assays were incubated in Pyrex tubes in a total volume of 100 μL with 2% v/v ethanol at 150 rpm and 35°C for 2 min. Afterwards, the protein was precipitated with 300 μL of methanol and formic acid to 1%. Conversions were analysed via HPLC-MS in triplicates. The specific activity was determined and the data was plotted with SigmaPlot.

### Effects of reaction conditions and N-glycosylation on Nh*KHS* activity

In order to investigate the effect of reaction conditions on *Nh*KHS activity, *in vitro* assays with 100 μg of purified protein and 0.5 mM XN in a final assay volume of 100 μL were performed. Unless otherwise mentioned, the assays were conducted on a shaker at 150 rpm and 28°C for 10 min. The effect of pH on *Nh*KHS activity was determined by performing *in vitro* reactions in buffers with varying pH values. The buffer systems used were 50 mM sodium citrate for pH 4.0–6.0, 50 mM potassium phosphate for pH 7.0–8.0, and 50 mM Tris-HCl for pH 9.0. The optimal reaction temperature was investigated by performing conversions from 15°C to 40°C in 5°C steps. The influence of N-glycosylation on *Nh*KHS activity was examined by *in vitro* deglycosylation of the enzyme with Endo*H*_*f*_ (Thermo Scientific, Austria). Therefore, 50 μL of *Nh*KHS with a concentration of 11 mg mL^-1^ were mixed with 0.5 μL of Endo*H*_*f*_ and 5.5 μL of G5 buffer (10x). Reactions were incubated for 0.5 to 2.5 h at 37°C. Control reactions contained 50 mM sodium citrate, pH 6.0, instead of Endo*H*_*f*_. SDS-PAGE was conducted by loading 2.5 μg of *Nh*KHS on the gel. In order to test for differences in stability and activity of (de-) glycosylated *Nh*KHS, thermal shift assays and *in vitro* activity assays with 20 μg and 100 μg of *Nh*KHS, respectively, were performed.

To detect the effect of organic solvents on the *Nh*KHS reaction, *in vitro* reactions with 0.05 mg mL^-1^ of purified enzyme and 2 mM XN were performed in the presence of 1%; 5%; 10% and 30% v/v of ethanol, DMSO, chloroform, dodecane and n-hexane, respectively. Conversions were incubated for 3 h at 35°C and 150 rpm.

### Thermal shift assay

The temperature stability of purified *Nh*KHS was determined by monitoring the fluorescence of a solvatochromic dye (ThermoFluor®) [21,22]. Therefore, SYPRO Orange (Thermo Scientific, Austria) was used as fluorescence indicator. Samples were prepared in 50 mM sodium citrate, sodium phosphate or Tris-HCl, ranging from pH 4.0 to 9.0 with a final concentration of 20 mM of either glycosylated or deglycosylated *Nh*KHS and addition of 2 μL of a SYPRO Orange solution (diluted 1:5000). Measurements were performed in triplicates with a CFX Connect Real-Time PCR system (BioRad, Hercules, CA). Samples were pre-heated to 25°C for 60 s before raising the temperature to 95°C in a 1°C min^-1^ ramp. The fluorescence emission of the SYPRO Orange dye was determined with a HEX fluorescence emission filter to monitor unfolding of the protein.

### HPLC analysis

Analyses were performed on an Agilent 1200 HPLC instrument equipped with MS and VWD detectors and a Poroshell HPLC column, (RP-18e 5 μm, 180 x 4.6 mm) maintained at 30°C. The elution system consisted of ddH_2_O with 0.1% formic acid (A) and acetonitrile with 0.1% formic acid (B). The gradient was set as follows: 0 min (40% B); 7 min (98% B); 8 min (5% B) at a flow rate of 0.7 mL min^-1^. KV, isoxanthohumol and 8-prenylnaringenin, as well as the respective reaction products were detected at a wavelength of 371 nm. XN was detected at 355.4 m/z and 371 nm and OH-XN at 373.4 m/z and 371 nm.

### Purification of XN from hop extract

XN was extracted from 600 g of hop extract (Hop extract capsules, Allcura, Germany) in three consecutive batches by adding 500 mL of methanol and 1 mL of formic acid to 200 g of hops extract. The mixture was heated to 60°C for 30 min. The insoluble solid was filtered off with a Büchner funnel and was washed four times with 75 mL each of hot methanol until the filtrate became virtually colorless. The combined solvent was removed under reduced pressure and the residue was stored at 4°C in the presence of nitrogen until continuing the work-up. The combined yield of extracts was 158.4 g. TLC analysis (cyclohexane/ethyl acetate 1:1) of the crude material yielded the following Rf values: 0.03–0.16 (most of contamination); 0.39 (XN); 0.79 and 0.85 (unknown compounds). For further identification of purity of the extract, a dilution of 1:100 was measured via HPLC UV 2.1 as described above. To remove polar contaminants from the extract, consecutive solid-phase extractions were conducted. The crude residue was treated with 400 mL of hot cyclohexane/ ethyl acetate (3:1) and the mixture was filtered through a 4 cm plug of approximately 50 g silica gel in a glass frit. Elution with cyclohexane/ethyl acetate (3:1) was continued until all XN was recovered. After removal of the volatiles under reduced pressure, 102.923 g of crude product were obtained. Then, 300 mL of hot acetone and 200 g silica gel were added and the solvent was removed to obtain the silica gel deposited crude material. This mass was placed on a flash chromatography column filled with 250 g silica gel pretreated with cyclohexane/ethyl acetate (4:1). Elution was started with 1 L cyclohexane/ethyl acetate (4:1) and then continued with cyclohexane/ethyl acetate (3:1). All fractions containing XN were combined and evaporated to dryness, leaving 32.985 g of crude product. This material was subjected to flash chromatography purification on 120 g silica gel using cyclohexane/ethyl acetate mixtures starting from 6:1 to 1:1 for elution. After removal of all volatiles from the fractions containing XN, 23.225 g of crude product was obtained. This product was sufficiently pure to continue with a recrystallization from approximately 45 mL of ethyl acetate/cyclohexane. Following a second recrystallization from 20 mL of ethyl acetate and 50 mL of cyclohexane, 4.23 g of pure XN were obtained (~ 99% purity; rp-HPLC at 210 nm). The mother liquors were combined and purified by flash chromatography on 200 g silica gel using dichloromethane/acetone (50:1) for elution. After pooling the product-containing fractions and removal of the volatiles under reduced pressure, 6.175 g of fairly pure XN were obtained. The material was recrystallized from 25 mL of ethyl acetate and 50 mL of cyclohexane leading to 4.938 g of pure XN (~ 99% purity; rp-HPLC at 210 nm). The overall yield of pure XN was 9.168 g.

### NMR analysis of the reaction product from XN

For confirmation of HO-XN formation from XN by ^1^H-NMR and ^13^C-NMR, *in vitro* conversions with a total amount of 5 mg of substrate were performed. One hundred μL of purified *Nh*KHS with a concentration of 1 mg mL^-1^ in 880 μL of 50 mM sodium citrate, pH 6.0, were incubated with 20 μL of 100 mM XN dissolved in ethanol. Seven bioconversion reactions were incubated in Pyrex reaction tubes in parallel on a shaker at 150 rpm and 35°C for 16 h. After addition of 3 mL of acetonitrile, the samples were centrifuged at 2,936 x g for 15 min and the product was extracted, dried and then dissolved in 4 mL of CD_3_OD for NMR measurement. ^1^H- and ^13^C- NMR spectra of XN isolated from hop extract and HO-XN obtained from *in vitro* conversions were measured on a Varian Inova-500 spectrometer (^1^H: 500 MHz; ^13^C: 125 MHz). Chemical shift values are reported in ppm (δ), and coupling constants (*J* values) are given in Hertz. Solvent was used as an internal standard. Abbreviations for ^1^H-NMR signals are as follows: s, singlet; d, doublet; t, triplet; dd, doublet of doublets; and m, multiplet. After NMR analysis, HO-XN was extracted and solidified.

XN:

^1^H-NMR (499.8 MHz, CD_3_OD): *δ* = 7.81 (d, J = 15.5 Hz, 1H, H-13), 7.69 (d, J = 15.5 Hz, 1H, H-14), 7.52 (d, J = 8.6 Hz, 2H, H-16), 6.85 (d, J = 8.6 Hz, 2H, H-17), 6.04 (s, 1H, H-7), 5.22 (t, J = 7.1 Hz, 1H, H-3), 3.92 (s, 3H, H-11), 3.25 (d, J = 7.1 Hz, 2H, H-4), 1.78 (s, 3H, H-1b), 1.67 (s, 3H, H-1a).

^13^C-NMR (125.7 MHz, CD_3_OD): *δ* = 192.7 (C-12), 164.8 (C-8), 162.3 (C-6), 161.0 (C-10), 159.6 (C-18), 141.9 (C-14), 130.0 (C-2), 129.8 (C-16), 127.1 (C-15), 124.5 (C-13), 122.9 (C-3), 115.5 (C-17), 108.0 (C-5), 105.2 (C-9), 90.3 (C-7), 54.8 (C-11), 24.5 (C-1a), 20.9 (C-4), 16.5 (C-1b).HO-XN:

^1^H-NMR (499.8 MHz, CD_3_OD): *δ* = 7.82 (d, J = 15.5 Hz, 1H, H-13), 7.71, (d, J = 15.5 Hz, 1H, H-14), 7.53 (d, J = 8.6 Hz, 2H, H-16), 6.93 (d, J = 8.6 Hz, 2H, H-17), 6.08 (s, 1H, H-7), 3.96 (s, 3H, H-11), 2.62 (t, J = 8.4 Hz, 2H, H-3), 1.66 (t, J = 8.4 Hz, 2H, H-4), 1.23 (s, 6H, H-1).

^13^C-NMR (125.7 MHz, CD_3_OD): *δ* = 192.8 (C-12), 164.8 (C-8), 162.4 (C-6), 161.0 (C-10), 159.6 (C-18), 142.0 (C-14), 129.8 (C-16), 127.1 (C-15), 124.5 (C-13), 115.5 (C-17), 108.7 (C-5), 105.2 (C-9), 90.4 (C-7), 70.4 (C-2), 54.8 (C-11), 42.0 (C-4), 27.6 (C-1), 17.1 (C-3).

## Results and discussion

### Searching for fungal kievitone hydratase sequences

To date, several studies have shown that detoxification of the plant isoflavonoid phytoalexins KV and phaseollidin by *F*. *solani* f. sp. *phaseoli* is conferred by at least two different enzymatic activities: Kievitone hydratase and phaseollidin hydratase [14]. Kievitone hydratase activity was also detected for other members of the *F*. *solani* species complex, such as *F*. *oxysporum* and *N*. *haematococca* by showing production of HO-KV from KV upon infection of the French bean [16]. Li et al. (1995) later detected sequences homologous to *Fs*KHS in different *N*. *haematococca* mating population (MP) VI isolates by Southern blot hybridization upon low stringency washing steps [15]. However, as of now no study could assign the enzymatic activity of *N*. *haematococca* to a specific gene or protein sequence. Therefore, we decided to search for sequences similar to *Fs*KHS in the four *N*. *haematococca* MPs that are listed in the BLAST suite on the NCBI web page (*N*. *haematococca* MP I, IV, V and VI) by using the pBLAST algorithm. Sequences with significant similarities were only found in *N*. *haematococca* MP VI 77-13-4, possibly due to the fact that it is one of the most extensively studied *Nectriaceae*, and the only one with a readily available genome sequence [23]. Upon search, five sequences produced significant alignments. Each represented a hypothetical protein with predicted hydroxyneurosporene synthase (CrtC) or peptidase activity without indication for kievitone hydratase activity. However, CrtC activity would, similar to a kievitone hydratase, imply the formation of a tertiary alcohol by addition of water to the non-activated terminal double bond of the 3-methyl-2-buten-1-yl moiety of the carotenoid neurosporene [24,25]. The DNA sequence (XM_003041138.1) corresponding to the hypothetical protein with the highest identity (58%) to *Fs*KHS was subsequently selected for heterologous expression in *P*. *pastoris* and *E*. *coli*.

The putative kievitone hydratase gene from *N*. *haematococca* (*Nh*KHS) codes for a peptide with 348 amino acid residues. Comparison of the protein and gene sequences with genome and nucleotide datasets did not reveal significant relationships to known proteins and genes from other organisms. A tblastn [19] analysis identified hypothetical proteins from *Aspergillus terreus* and *Aspergillus nidulans* as the closest relatives, sharing a sequence identity of only 43% and 35%, respectively, with *Fs*KHS. Despite an overall low sequence identity between the tblastn alignments of the four proteins with the highest similarity, a Clustal Omega alignment revealed a number of conserved amino acid residues among the selected sequences (S1 Fig). Interestingly, the *N*-terminal region of the alignment showed a noticeable variation in length and contained only few fully or strongly conserved residues. However, in the middle and *C*-terminal parts, a number of conserved residues frequently appeared over the whole segment of the sequence. Provided that all four amino acid sequences show kievitone hydratase activity, it can be argued that highly conserved residues located in these regions may be of high importance for either substrate binding or the catalytic mechanism.

A 19 amino acid long signal peptide absent from the predominantly secreted mature enzyme was identified for *Fs*KHS with a cleavage site between alanine 19 and serine 20 [15,26]. Similarly, according to SignalP, the cleavage site of the putative *N*-terminal signal peptide of *Nh*KHS is predicted between alanine 19 and lysine 20 [27]. While the *N*-terminal part of *Nh*KHS is in accordance with the rules proposed by van Heijne [28] the signal sequence of *Fs*KHS does not include basic amino acids in its *N*-terminal region [23].

Further analyses did not indicate the presence of any nucleotide cofactor due to the absence of a conserved nucleotide binding motif [29,30]. However, aside from the cleavage of a putative *N*-terminal signal sequence, the protein was expected to be N-glycosylated, as four typical consensus sequences for N-linked glycosylation were found [31]. Extensive glycosylation was shown previously for kievitone and phaseollidin hydratases from *F*. *solani* f. sp. *phaseoli* [13,14].

### Expression and purification of *Nh*KHS

Protein expression studies were conducted in *E*. *coli* and *P*. *pastoris*. Using *E*. *coli* as a host for expression of *Nh*KHS after codon-optimization, extensive formation of inclusion bodies without yielding notable amounts of soluble protein was detected by SDS-PAGE and Western blotting. Therefore, *E*. *coli* was not further regarded as a host for expression of *Nh*KHS.

Subsequently, the *C-*terminally His_10_-tagged *Nh*KHS gene was expressed in the methylotrophic yeast *P*. pastoris. The gene was codon-harmonized and cloned into the expression vectors p*Pp*T4_S and p*Pp*T4_Alpha_S [17] via *Eco*RI and *Hind*III restriction sites (S2 Fig). Initial analyses showed the best secretory expression using the vector p*Pp*T4_Alpha_S. With the p*Pp*T4_S vector, secretory expression was notably worse, whereas *Nh*KHS expression was even more severely affected when lacking the *N*-terminal part coding for the putative signal sequence. Simultaneously, the gene coding for C-terminally His_10_-tagged *FsKHS* was cloned into p*Pp*T4_S and p*Pp*T4_Alpha_S and expressed under the same conditions as *Nh*KHS. However, upon recombinant expression in *P*. *pastoris*, protein levels of *Fs*KHS in the culture supernatant were clearly lower than those obtained for the strain expressing *Nh*KHS. Therefore, all further studies were performed using *PpKHS*Alpha. Only a minor fraction of cell-associated *Nh*KHS was detected by Western blotting, indicating highly efficient secretion of the enzyme by *P*. *pastoris*. Analysis of protein levels in the culture supernatant after precipitation with the chloroform/methanol method revealed a band at an estimated size of ~ 48 kDa on SDS-PAGE. This indicated that the protein was notably larger than the calculated size of 38 kDa of the mature polypeptide, which was attributed to glycosylation at one or several of the four N-glycosylation sites [13,14].

**Fig 1:**
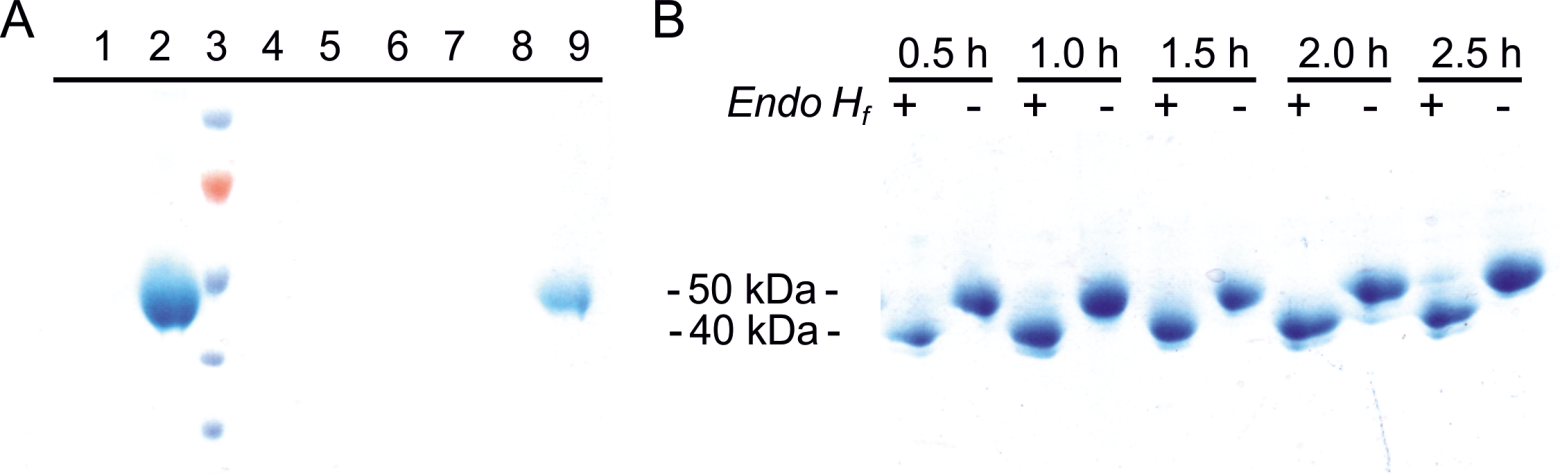
**Analysis of secreted protein and purification of *Nh*KHS from *P*. *pastoris* culture supernatant (A).** Proteins were precipitated from the culture supernatant of *P*. *pastoris* wild type strain (lane 1) and *PpKHS*Alpha strain (lane 2) with the chloroform/methanol method. Four hundred **μ**L of cell culture were precipitated. KHS was purified via Ni-NTA affinity chromatography. Ten **μ**L of flow through (lane 4), washing fractions with 10 mM imidazole (lanes 5 and 6) and 50 mM imidazole (lanes 7 and 8), as well as 4 **μ**L of concentrated, pooled elution fractions (lane 9) were loaded onto the gel. PageRuler^TM^ Prestained Protein Ladder was used as molecular weight standard (Lane 3). ***In vitro* deglycosylation of purified KHS with Endo*H***_***f***_ **(B).** Deglycosylation reactions were incubated for 0.5–2.5 h.

Due to the low levels of secreted endogenous proteins in *P*. *pastoris*, *Nh*KHS could already be partially purified to a good extent by secretion of the enzyme into the culture supernatant. His_10_-tagged *Nh*KHS was further purified from the culture supernatant using self-packed Ni-NTA columns. Affinity chromatography resulted in isolation of highly pure *Nh*KHS that was used for further biochemical analyses (Fig 1A). Upon *in vitro* deglycosylation with Endo*H*_*f*_, the apparent molecular weight was matching the calculated size of the non-glycosylated enzyme of 38 kDa (Fig 1B). Gel filtration of His_10_-tag purified *Nh*KHS on a Superdex 200 HiLoad 16/60 column resulted in detection of one single peak at an elution volume of 77 mL (S3 Fig). On the basis of a calibration with standard proteins, a molecular weight of approx. 75 kDa was determined for *Nh*KHS. This observation suggests that the native form of the enzyme in solution is most likely a homodimer. UV-Vis absorption spectral analyses of the purified enzyme did not indicate the presence of any chromophores in the range from 300 to 1,000 nm, suggesting the absence of any prosthetic group.

### *In* vitro conversion of KV with recombinantly expressed *Nh*KHS

**Fig 2:**
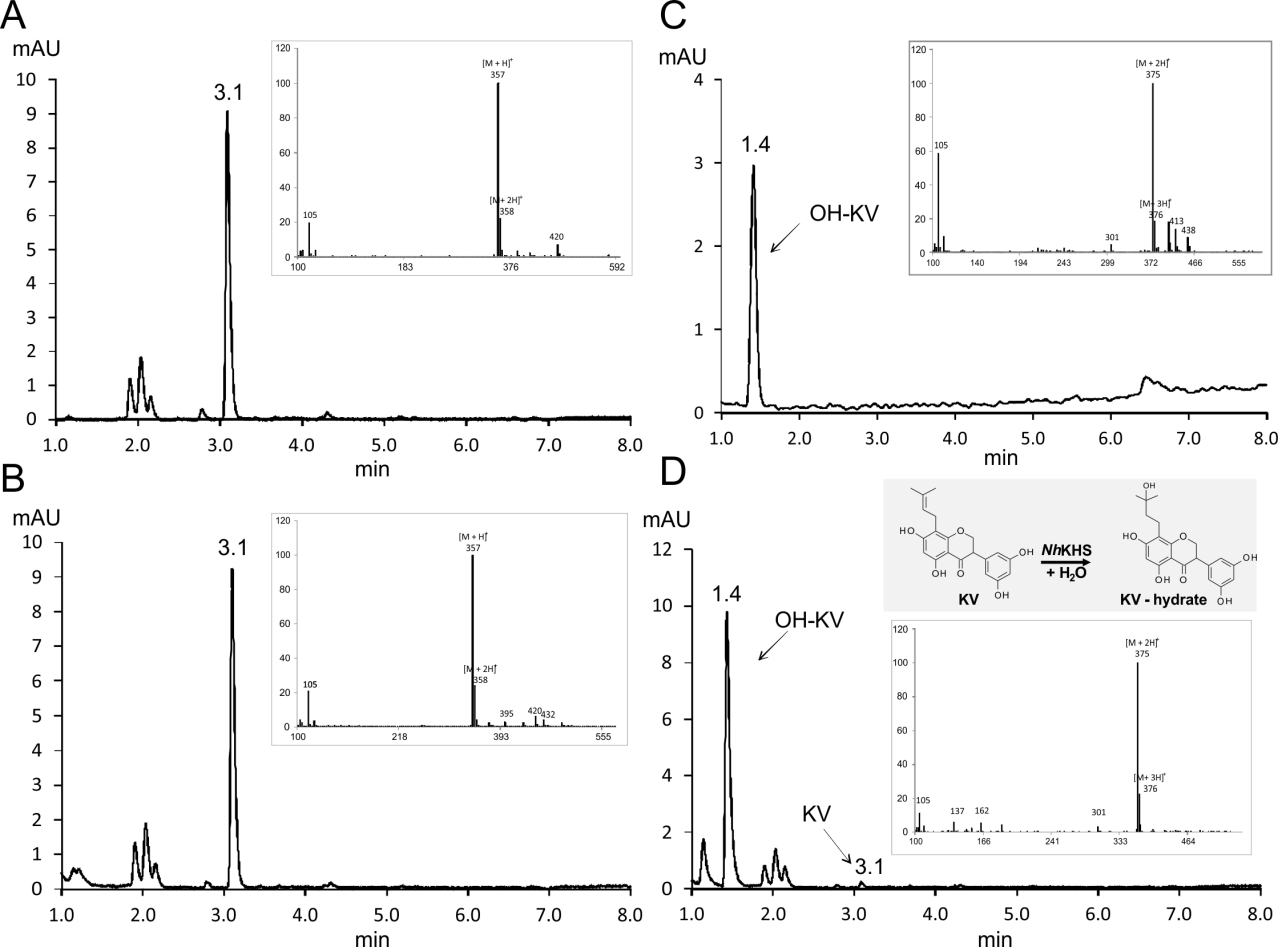
Formation of HO-KV from KV catalyzed by *Nh*KHS. *P*. *pastoris* culture supernatants were incubated with KV for 3 h at 35°C and product formation was analyzed via HPLC-UV at 371 nm. Insets represent mass spectra of selected peaks at respective retention times determined by HPLC-MS in positive SIM mode. The retention time of the substrate was determined by analysis of a 2 mM authentic KV standard (A). No product formation was observed using the culture supernatant of a *P*. *pastoris* WT control (B). Inset of (A & B) represent the mass spectrum of the KV peak at a retention time of 3.1 min. The retention time of the product was determined by analysis of a 0.5 mM authentic HO-KV standard (C). HO-KV was formed using the culture supernatant of *PpKHS*Alpha expressing *Nh*KHS (D). Insets of (C & D) represent the mass spectrum of the product peak at a retention time of 1.4 min.

Functional expression of *Nh*KHS was validated by hydratase activity measurements with *P*. *pastoris* culture supernatant containing ~ 0.6 mg mL^-1^ of recombinantly expressed protein. In order to test whether the putative KHS from *N*. *haematococca* showed activity for the isoflavanon KV, *Nh*KHS was incubated with 2 mM KV in 2% ethanol (v/v) for 3 h. *In vitro* reactions were analyzed by HPLC-UV. Retention times and mass fragmentation patterns of substrate and product in conversions were compared with authentic KV and HO-KV standards (Fig 2A and 2C). *In vitro* conversions with *PpKHS*Alpha and KV as substrate resulted in formation of one additional peak, as well as the consumption of approx. 91% of the substrate (Fig 2D). No putative product peak was formed in the control reaction with *P*. *pastoris* WT culture supernatant (Fig 2B). Furthermore, mass fragmentation patterns of substrate and product were identical with the ones obtained for the authentic standards (Fig 2, insets). Thus, formation of HO-KV was confirmed and the enzyme from *N*. *haematococca* did indeed show KHS activity. Additionally, *Fs*KHS in *P*. *pastoris* culture supernatant was used to convert KV *in vitro*. Despite a markedly lower protein expression level than *Nh*KHS, approx. 89% of the substrate was converted by *Fs*KHS in the culture supernatant within 3 h of incubation.

### Substrate scope of *Nh*KHS for different flavonoids

**Fig 3:**
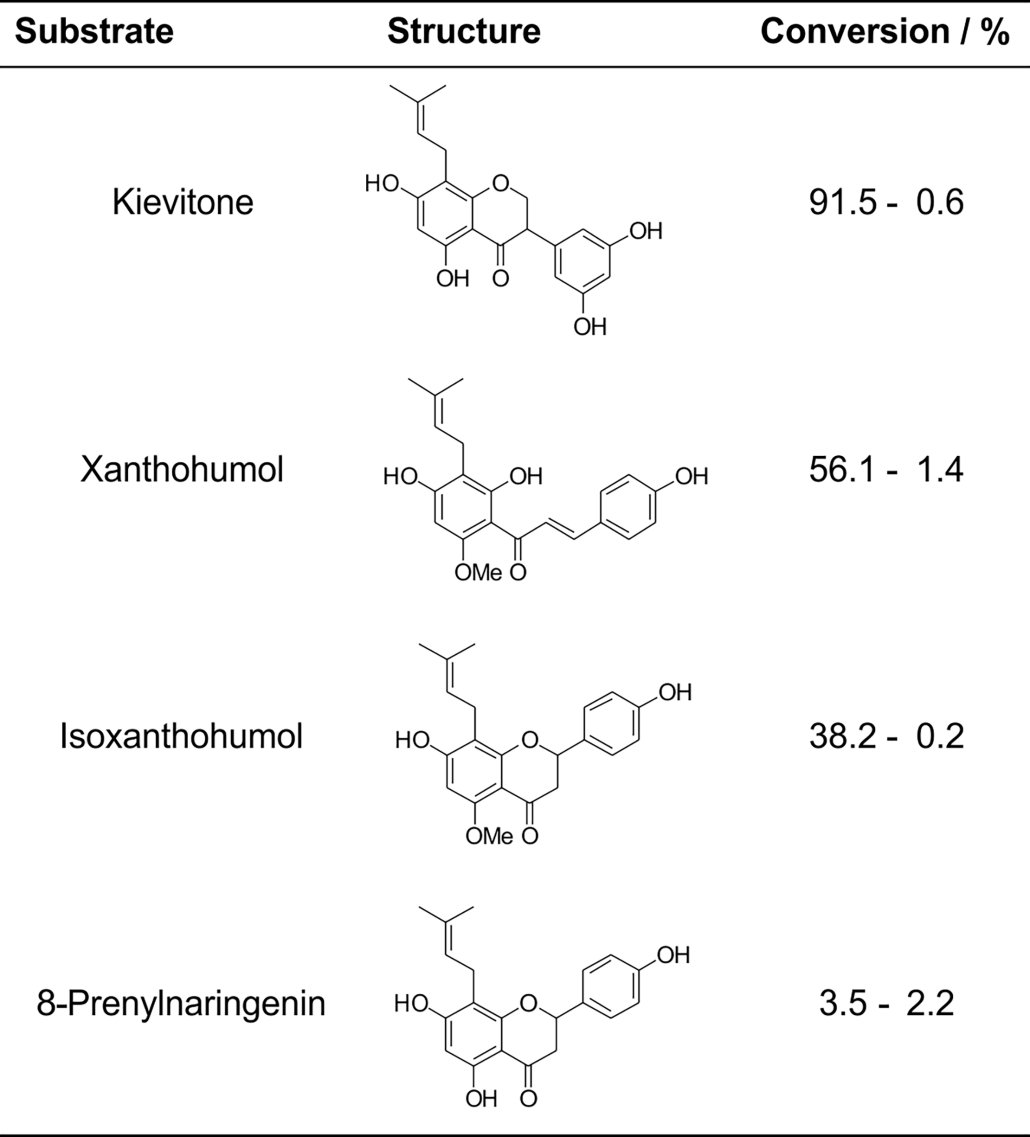
Flavonoid substrates converted with *Nh*KHS.

Since KV and phaseollidin are commercially not/rarely available, further biochemical and functional studies were initially restricted. Therefore, identification of alternative substrates for further analyses was highly desirable. The more easily accessible flavonoids 8-prenylnaringenin and isoxanthohumol, as well as the chalcone derivative xanthohumol (XN) were purchased and used for conversion reactions with *Nh*KHS (Fig 3). Selection criteria for potential alternative substrates were a close similarity of their prenylated A-ring core structure with KV, as well as differently attached B-rings on the pyran-moiety of the C_15_ flavan scaffold in order to assess potential effects thereof on enzyme activity. Whereas the B-ring of the isoflavanon derivative KV is attached at C3, the B-ring of the prenylated flavanons 8-prenylnaringenin and isoxanthohumol is attached at C2. In contrast, XN lacks the typical flavonoid structure due to its chalconoid skeleton. Furthermore, the selected compounds differed in their hydroxyl- and methoxy-substituents, respectively.

**Fig 4:**
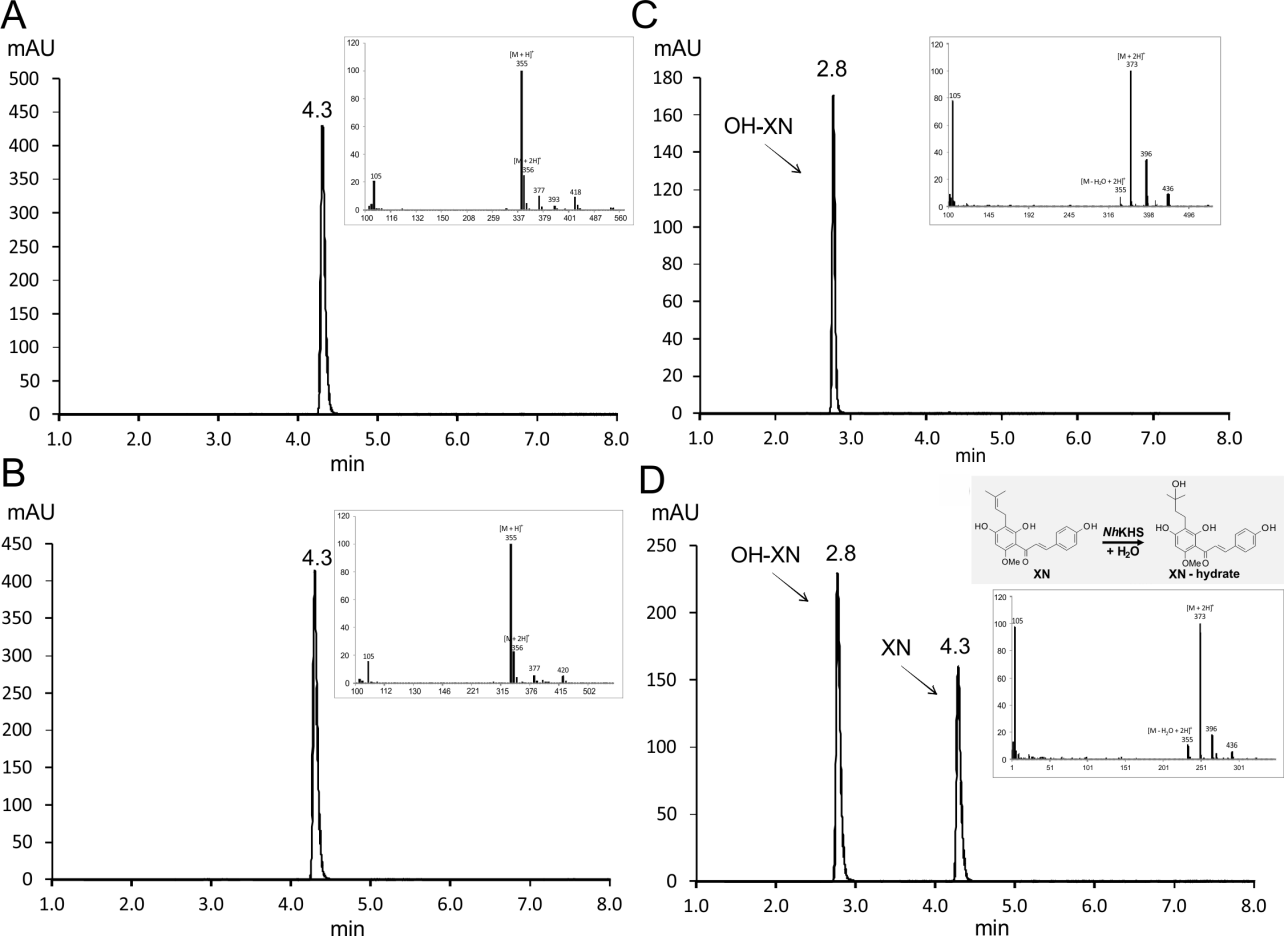
Formation of HO-XN from XN catalyzed by *Nh*KHS. *P*. *pastoris* culture supernatants were incubated with XN for 3 h at 35°C and product formation was analyzed via HPLC-UV at 371 nm. Insets represent mass spectra of selected peaks at respective retention times determined by HPLC-MS in positive SIM mode. The retention time of the substrate was determined by analysis of a 2 mM authentic XN standard (A). No product formation was observed using the culture supernatant of a *P*. *pastoris* WT control (B). Insets of (A & B) represent the mass spectrum of the XN peak at a retention time of 4.3 min. The retention time of the product was determined by analysis of a 0.5 mM authentic HO-XN standard (C). HO-XN was formed using the culture supernatant of *PpKHS*Alpha expressing *NhKHS* (D). Insets of (C & D) represent the mass spectrum of the product peak at a retention time of 2.8 min.

All tested flavonoids (Fig 3) were converted by recombinantly expressed and secreted *Nh*KHS as described in Materials and Methods. The highest conversion was obtained for kievitone (91.5 ± 0.6%), followed by XN (56.1 ± 1.4%, see Fig 4), isoxanthohumol (38.2 ± 0.2%) and 8-prenylnaringenin (3.5 ± 2.2%). These results underscore that the enzyme is indeed a KHS with a substantial degree of substrate promiscuity. The lower activity for 8-prenylnaringenin and isoxanthohumol compared to XN may be a consequence of the differently attached B-ring in comparison to KV, resulting in a more pronounced steric hindrance of substrate binding in the cavity. This restriction may be less of a problem in the case of XN, since the chalconoid scaffold resembles the shape of the isoflavanon KV more closely. Since the only common moieties of all tested compounds are the aromatic A-ring and the hydroxyl-substituent at C7, it may be speculated that substrate binding to the active site is mediated by either π-π stacking of the A-ring with aromatic amino acid residues of *Nh*KHS, or interaction of polar side chains with the hydroxyl group at C7, or both.

### Isolation of XN from hop extract and NMR analysis of the reaction product

Owing to the good conversion and easy access to XN, we selected it as a model substrate for biochemical analysis of *Nh*KHS. Sufficient amounts of XN were obtained by extraction from commercially available hop extract and subsequent purification. The final yield of XN was approximately 9.2 g with a purity of 99% as shown by HPLC (Fig 4B). NMR analysis of isolated XN and the reaction product after incubation of XN with 1 mg mL^-1^ of purified *Nh*KHS unambiguously confirmed hydration of XN [32] to HO-XN [33] (S4 Fig).

### Enzyme kinetics

**Fig 5:**
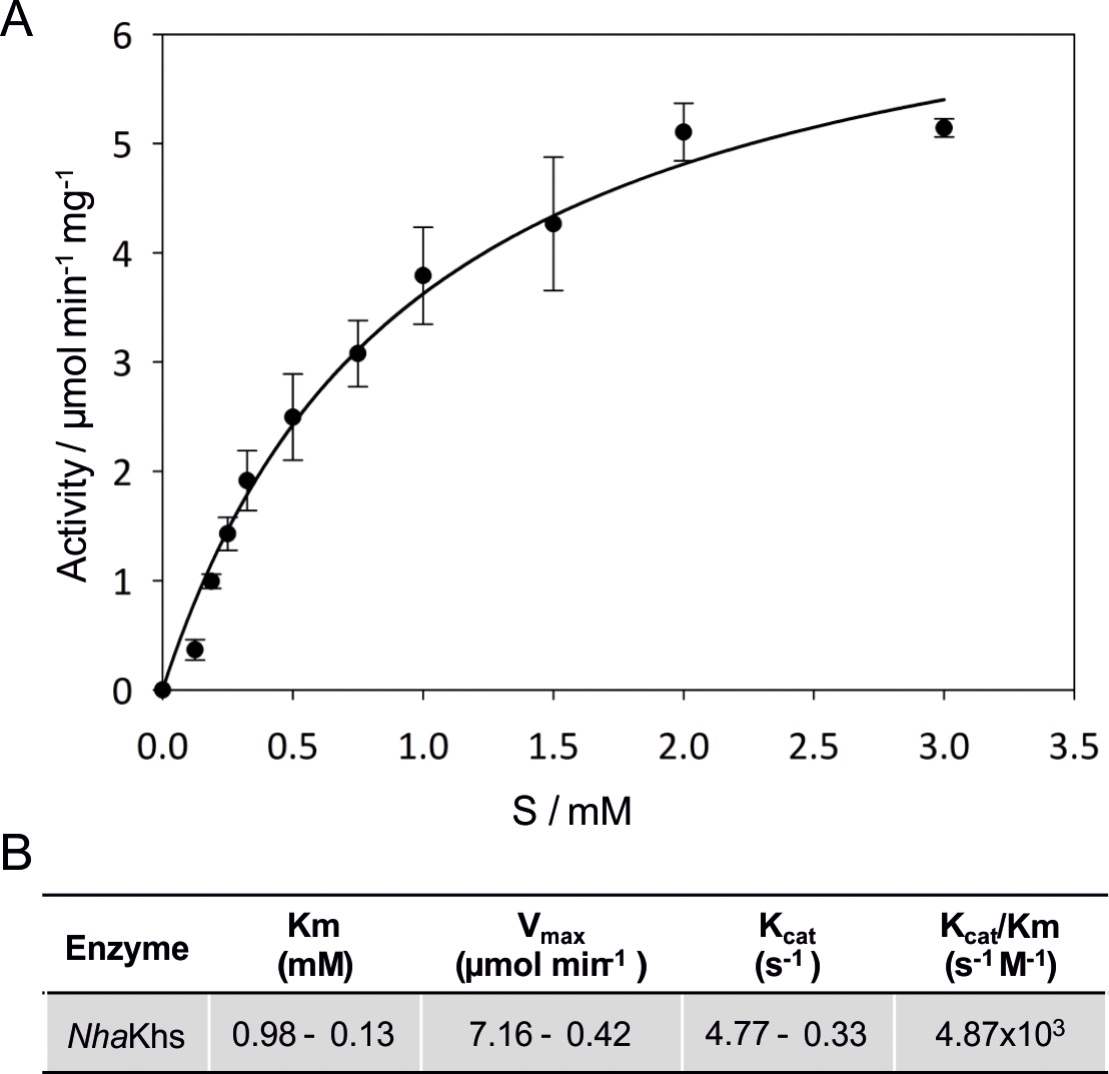
Kinetic characterization of *Nh*KHS with the substrate XN. Reaction mixtures contained 0.05 mg mL^-1^ of purified enzyme in 50 mM sodium citrate, pH 6.0, with 2% ethanol (v/v) and the XN concentration was varied from 0.125 to 3.0 mM. Reactions were incubated for 2 min at 35°C and 150 rpm in triplicates. (A) Michaelis-Menten plot. Kinetic parameters were determined directly from the regression data of a nonlinear hyperbolic curve fit. (B) Summary of kinetic constants for XN hydration by *Nh*KHS.

Kinetic assays were performed *in vitro* under optimized reaction conditions. Kinetic parameters were determined with XN concentrations ranging from 0.125 to 3.0 mM. Due to the low solubility of XN in the aqueous buffer system, even upon addition of 2% ethanol (v/v) as co-solvent, only apparent values for the substrate concentration, as well as V_max_ and K_m_ can be stated. Results of kinetic measurements are shown in a Michaelis-Menten plot as the initial reaction rate versus the apparent substrate concentration (Fig 5). K_m,app_ and V_max,app_ of recombinant *Nh*KHS were calculated as 0.98 ± 0.13 mM and 7.16 ± 0.42 μmol min^-1^ mg^-1^, respectively, with a k_cat_ of 4.77 ± 0.33 s^-1^ and a catalytic efficiency of 4.87 x 10^3^ s^-1^ M^-1^. Our data represent the first enzyme kinetics for the activity of a KHS for a non-physiological substrate. Until now, only *Fs*KHS was kinetically characterized, with values for V_max,app_ and K_m,app_ for kievitone of 4.92 nmol min^-1^ mL^-1^ (0.21 μmol min^-1^ mg^-1^) and 0.0175 mM, respectively [13].

### Effect of reaction conditions and glycosylation on enzyme activity and stability

**Fig 6:**
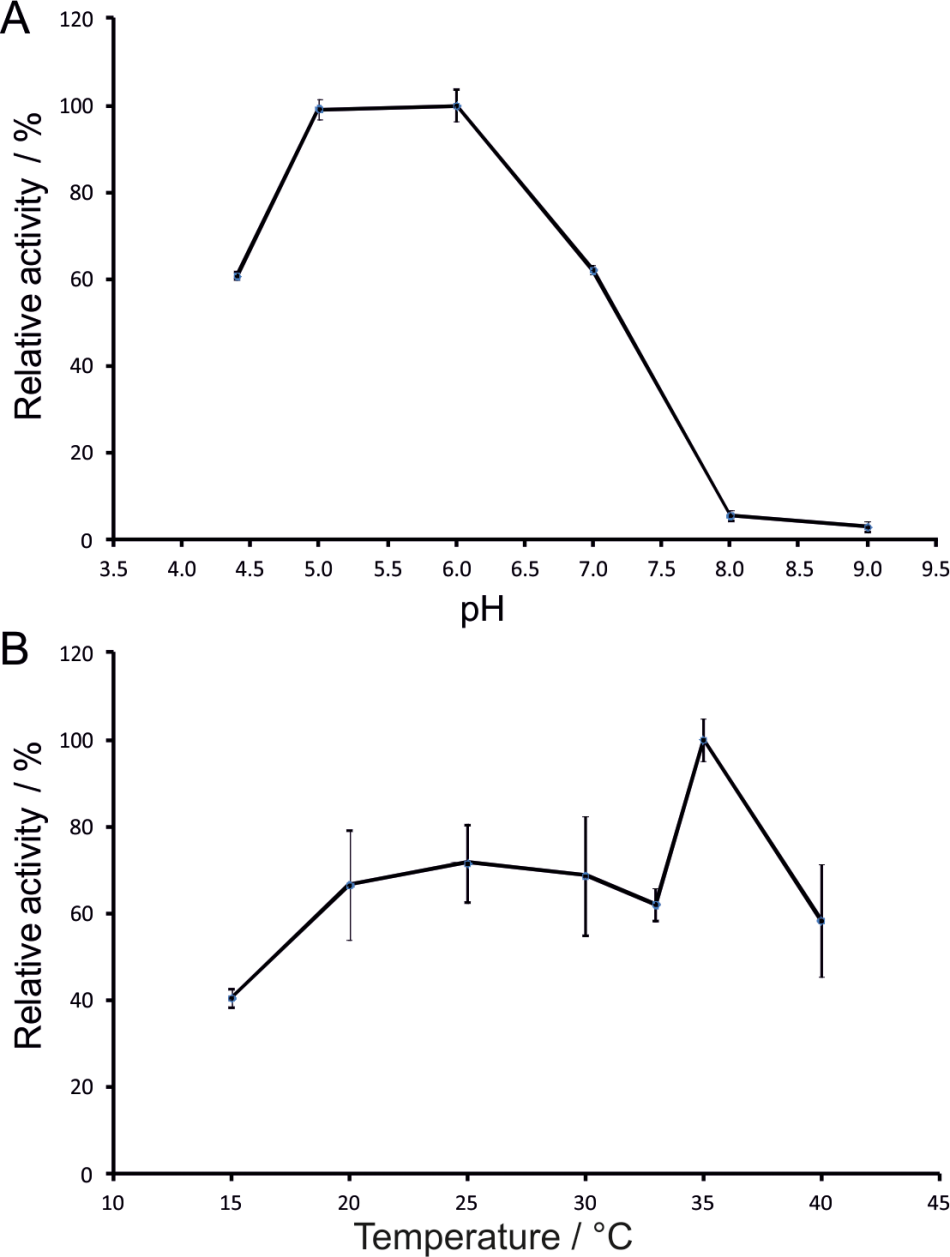
Effect of reaction conditions on the conversion of XN by *Nh*KHS. For determination of reaction optima, 100 **μ**g of purified enzyme were incubated with 0.5 mM substrate for 10 min at 28°C in triplicates. (A) The effect of pH on *Nh*KHS activity was investigated in 50 mM sodium citrate at pH 4.5–6.0, 50 mM potassium phosphate at pH 7.0–8.0 and 50 mM Tris-HCl at pH 9.0. (B) The effect of temperature on enzyme activity was determined by incubation of reactions at 15°C- 40°C.

Using XN, we investigated the influence of pH and temperature on the activity and stability of recombinant *Nh*KHS. The favorable pH range for hydratase activity was between pH 5.0 to 6.0 (Fig 6A). Around 60% of the relative activity was remaining at pH values of 4.5 and 7.0, whereas raising the pH to 8.0 resulted in a rapid decrease of the activity below 10%. This indicated that the enzyme was relatively stable in a slightly acidic environment but was rapidly inactivated in alkaline conditions. According to the pH curve reported for *Fs*KHS by Cleveland and Smith, the optimal pH for enzyme activity was 5.5 and pH values above 7.5 resulted in a decrease of the activity below 10% [13]. Therefore, despite notable differences between both enzymes on the sequence level, the pH optima were similar. Furthermore, the optimal pH for *Nh*KHS activity was in good agreement with overall enzyme stability as shown by thermal shift assays with the respective buffers from pH 4.0 to 9.0. The highest T_m_ of 50.3 ± 0.6°C was detected at pH 6.0. Similarly to the effect on activity, slightly acidic pH values only had a minor effect on overall enzyme stability, whereas the T_m_ at pH 8.0 decreased markedly to 15 ± 0.1°C (S2 Table). The effect of temperature on hydratase activity was assessed from 15°C to 45°C. Among the tested reaction conditions, the best conversion was obtained at a temperature of 35°C (Fig 6B). Reactions at slightly lower and higher temperatures resulted in a substantial decrease of the relative activity. However, further reduction of the reaction temperature only had a minor additional impact on the conversion. Since for *Fs*KHS an optimal reaction temperature of 55°C was reported, temperature optima of different KHS appear to differ substantially [13].

So far, all known hydratases conferring the detoxification of plant flavonoid phytoalexins were found to be extensively N-glycosylated [13,14]. Observation of a high temperature optimum for *Fs*KHS suggested that glycosylation might play a role in heat stability of the enzyme. However, the function of N-glycosylation for KHS has not been experimentally tested so far. Therefore, we analyzed potential effects of glycosylation on enzyme stability and activity by comparing glycosylated and deglycosylated *Nh*KHS in thermal shift assays and by *in vitro* conversion of XN. The temperature stability of the enzyme was apparently not affected by deglycosylation as indicated by highly similar T_m_ values of both enzyme forms at pH values ranging from pH 4.0 to pH 9.0 (S2 Table). In contrast, *in vitro* activity assays showed that the activity of the deglycosylated enzyme was approx. 30% lower compared to the glycosylated control. Thus, our results suggest a role of N-glycosylation for enzyme activity, but not for overall stability as speculated previously. However, additional functional analyses in consideration of structural information—once available—will still be necessary to determine the role of N-glycosylation in enzyme function in more detail.

The effect of organic solvents on enzyme activity was investigated by incubation of purified *Nh*KHS with XN in the presence of 1–30% v/v of different solvents. After 3 h of incubation, the highest activity was retained with hexane and n-dodecane. In these cases, addition of up to 30% v/v of solvent to the reactions did not result in notably reduced product formation compared to the control (S5 Fig). However, product formation was already considerably affected in the presence of 10% v/v of ethanol, DMSO or chloroform. This indicates that the polarity of the organic solvent is a determining factor for its effect on *Nh*KHS activity, with increasing solubility in water resulting in a more distinct impact. This might be caused by an enhanced perturbing effect on the hydration shell of the enzyme. However, the compatibility of *Nh*KHS with nonpolar organic solvents will be beneficial for a number of biocatalytic applications, e.g. in general for conversion of hydrophobic substrates.

### Bioreactor cultivation

An outstanding feature of the *P*. *pastoris* host system for recombinant protein expression is its ability to grow to high cell densities of more than 150 g CDW L^-1^ in controlled fed-batch cultivations [34]. Since this often results in high yields of recombinant protein, we tested whether scaling up protein expression from shake flask to lab scale fed-batch fermentation is a feasible strategy to increase the production of recombinant *Nh*KHS. Therefore, *PpKHS*Alpha and *P*. *pastoris* wild type strains were cultivated in parallel in 2 L bioreactors (DASGIP, Jülich, Germany). At different time points of methanol induction, culture aliquots were withdrawn for determination of CDW, protein concentration, expression level and enzyme activity. At the end of induction, CDWs of the different strains were similar, reaching values in the range of 80–90 g L^-1^ of culture. SDS-PAGE confirmed that protein levels increased steadily during induction (S6 Fig). At the end of the cultivation, 2.61 g L^-1^ of secreted protein was obtained from 2 L of fermentation broth. Comparison of CDW and protein levels in the culture supernatant of bioreactor (89 g L^-1^ CDW; 2.3 g L^-1^ of recombinant *Nh*KHS) and shake flask cultivation (9.6 g L^-1^ CDW; 0.6 g L^-1^ of recombinant *Nh*KHS) under standard conditions, i.e. 400 mL of medium, 48 h of methanol induction, indicated that the notably higher yield of *Nh*KHS in the bioreactor cultivation indeed resulted from the higher cell density in the bioreactor. Overall, *P*. *pastoris* is a most appropriate host for producing high amounts of recombinant *Nh*KHS.

In summary, our study reports on the identification and heterologous expression of KHS from *N*. *haematococca* MP VI that catalyzes the formation of a tertiary alcohol by hydration of the phytoalexin KV. After investigating into the substrate spectrum of KHS, the first thorough biochemical characterization of a KHS was performed by using XN as the model substrate. Since *Nh*KHS showed good activity for a range of different non-physiological substrates as well as stability at broad and suitable temperature and pH ranges, it is a promising candidate for the biosynthesis of industrially relevant tertiary alcohols.

## Supporting information

S1 Fig Clustal Omega alignment of putative KHS enzymes. Protein sequences from *A*. *nidulans*, *A*. *terreus*, *F*. *solani* and *N*. *haematococca* are shown.
(PDF)

S2 Fig Coding sequence of codon-harmonized putative kievitone hydratases from *N*. *haematococca* (*Nh*KHS) and from *F*. *solani* (*Fs*KHS) for expression in *P*. *pastoris*. (PDF)

S3 Fig Gel filtration profile of *Nh*KHS on a Superdex 200 HiLoad 16/60 column (GE Healthcare, UK) using 50 mM sodium citrate, pH 6.0, as buffer system. Inset: Calibration curve generated with the standard proteins conalbumin (75 kDa), ovalbumin (44 kDa), carbonic anhydrase (29 kDa), ribonuclease A (13.7 kDa) and aprotinin (6.5 kDa).
(PDF)

S4 Fig ^1^H- and ^13^C-NMR spectra of XN and HO-XN in CD_3_OD. After incubation of XN with purified *Nh*KHS, the reaction products were extracted and prepared for NMR analysis as described. (A) ^1^H-NMR spectrum of XN at 499.8 MHz. (B) ^13^C-NMR spectrum of XN at 125.7 MHz. (C) ^1^H-NMR spectrum of HO-XN at 499.8 MHz. (D) ^13^C-NMR spectrum of HO-XN at 125.7 MHz. HO-XN could be unambiguously identified as the reaction product upon hydration of XN by *Nh*KHS, and substrate and product were independently confirmed by comparison with ^1^H- and ^13^C-NMR spectra provided in other work [1,2].
(PDF)

S5 Fig Influence of different organic solvents on the activity of *Nh*KHS. The standard enzyme assay was performed in the presence of respective organic solvents at concentrations ranging from 0.5 to 30%. One mM of XN and 0.05 mg mL^-1^ of enzyme were incubated for 3 h. Amounts of XN-hydrate in mM were obtained via HPLC-MS measurements. Biological triplicates were analyzed.
(PDF)

S6 Fig Bioreactor cultivation of strain *PpKHS*Alpha. Results of activity assays using 3 μL of supernatant of the fermentation broth from strain *PpKHS*Alpha at indicated time points of induction (A). Volumetric activity of strain *PpKHS*Alpha (B). Activity assays and HPLC analyses were performed in triplicates. *Nh*KHS levels in culture supernatants were monitored by SDS-PAGE (C) and compared to purified protein (KHS pur.).
(PDF)

S1 Table Primers used for cloning of *Nh*KHS and *FsKHS* into the vector p*Pp*T4_Alpha_S. (PDF)

S2 Table Thermostability of glycosylated and deglycosylated *Nh*KHS at different pH values. Purified enzyme was deglycosylated with Endo*H*_*f*_ enzyme mix for 0.5 h at 37°C.
(PDF)
