# Supplementary material for: Recombinant expression, purification and biochemical characterization of kievitone hydratase from Nectria haematococca
Source: PLoS One. 2018 Feb 8;13(2):e0192653. doi: 10.1371/journal.pone.0192653 (PMC5805349; doi:10.1371/journal.pone.0192653)
Supplement: S1 Fig — Protein sequences from A. nidulans, A. terreus, F. solani and N. haematococca are shown. (PDF) [file pone.0192653.s001.pdf]

```
A. nidulans   ------------------------MRP--AILAAFSTLP-------AAAKATYPFAPETF
A. terreus    MDIARGVLLWALTQVLYEIPEQVSMQFGSRVIRPRPRIPPCTHIPLPSRGRRNILAGKST
FsKHS         ------------------------MMISSVLVAGVVAVSAALA---SKHPKQYSFKPEDA
NhKHS         ------------------------MRASFLLTAGLATAAVGRA---KSVPKKFPFKPENS
                                      *     :                       :  :

A. nidulans   DGSYKDIGLPTIYDLSATQ---STNYNGSWATGSWITSVSGGQYFVVSHYVNDGI-HDVY
A. terreus    TPA-LNPSTPLLFNFSHSKLLNTTSSTNSYWTSSYITNTTNEQYLLISHLLVGPSDRSWY
FsKHS         ETI-WNGDIPVLYDFGDSQ---SASYSGSWWTSSYITGTNGEQYLVISHYLDTPV-FTYF
NhKHS         KTT-GTNAIPIVYGLSESQ---PNSVGGSWWSSSYITTTNNEQYVVLAHYLDNPV-YTYF
                       * ::.:. ::     .  .*: :.*:** ... **.:::* :        :

A. nidulans   RSSILDLSSLKYRYFFQAGNGSYTASPPSHLKAGVGKGNGFEGISNDNYTTMRVQ-SSHP
A. terreus    RASALNLANPSNYTT-TVTSSTAP----------VF-QPGP-----QLRATMRTFSADSP
FsKHS         RASTLNLETLDYNQFITLGNNTAN---STTLDVKVG-DNGIQSLTADNISQQRAY-ANDE
NhKHS         RASTLNLETNEYHQYVTVGSSTPN---ITTLDVSVG-NNGIKSESEDNLSKLRSY-SNHD
              *:* *:* . .        ..:            *    *      :  :  *   :.

A. nidulans   NVTFDLTYHATTKPLINGGAGVVMLGASESKQWSLPACWTNGFLIVG-DEQIPIDPKRSL
A. terreus    VLTFDLTYAATSTALVNGGMGLFAFGSGRTYEWGLPNCVTTGSARIN-GAPLRVDPARSF
FsKHS         NVTFDITFDATSRVISNAGAGVFQFGPSITYEWGLPNCRTQGSVTDTGGKNITVDPAKSF
NhKHS         NVTFDITYDATTGAVANGGAGTFQFGEGLTWEFGLPSAKTEGSLTVH-GEKLAIDPAKSH
               :***:*: **:  : *.* * . :* . : ::.** . * *      .  : :** :*

A. nidulans   TWYDRQWGTGGL--TNWTWYGLHIPKTGHVLSIWTGDTDADRAAPITPVRFATVRNAYGA
A. terreus    TWFDRQWIEAAA-AASWTRFELHVAGSRDRLSVWAIDSGEGG---GPGVRFATIRRGDGA
FsKHS         TWYDRQWGTAAVTSGNWTWFQMHIPETSYKLSVWIIDNDV-T---NQFSRFATIRGDNDE
NhKHS         TWYDRQWGNTAAIPSNWTWFQLHIPSTEYKISAWIFSDPF-R---NTETRFATIRGANDE
              **:****   .    .** : :*:  :   :* *  .            ****:*   .

A. nidulans   QTVCNITWIPDLSHIFHSDSTNKSYPLAWTVEIPSYDAIIKVKSRTENQLNTGSHGSEPE
A. terreus    QLVVPVGFRADYTRQWYSQASGKLYPLDWVVTVGEVGVF-RIASIVGDQEIAG-GSAFPT
FsKHS         FQVLPLEWKPIYDRTYQSTAADILYPLDWELDISGFGVF-QLSSILDDQEIVG-TTAIQT
NhKHS         TLVLPLEFTPIYKRTYESATGRVTYPLDWKLKISGFGDF-KLSSYTEDQELVG-EDALQT
                *  : :     : : * :    *** * : :   . : :: *   :*  .*   :

A. nidulans   AYNGFVTFAGQFQGTETEGFGIVEIVYL-----    347
A. terreus    AYEGFVTFDGRFEGQEVGGFGVVEIIFSGES--    366
FsKHS         AYEGFVTFNGTVHNKKVQGYGLVEVVYSNWESL    350
NhKHS         AYEGFITFSGNVHSKPVQGYGLVEIVYSTWDV-    348
              **:**:** * ...  . *:*:**:::
```

S1 Figure. Clustal Omega alignment of putative KHS enzymes. Protein sequences from *A. nidulans*, *A. terreus*, *F. solani* and *N. haematococca* are shown.
