# Supplementary material for: Recombinant expression, purification and biochemical characterization of kievitone hydratase from Nectria haematococca
Source: PLoS One. 2018 Feb 8;13(2):e0192653. doi: 10.1371/journal.pone.0192653 (PMC5805349; doi:10.1371/journal.pone.0192653)
Supplement: S2 Fig — (PDF) [file pone.0192653.s002.pdf]

>CDS_*Nh*KHS

ATGAGAGCTTCCTTTCTTCTGACCGCCGGTTTGGCTACCGCAGCCGTGGGACGTGCTAAGTCTGTGCCTAA
GAAATTTCCTTTCAAGCCAGAAAATTCTAAGACCACAGGAACAAATGCAATTCCCATAGTATATGGCCTGAGTG
AATCCCAACCAAATTCTGTCGGTGGATCTTGGTGGTCATCTTCCTATATCACTACCACTAATAACGAACAATACG
TCGTTTTGGCTCATTATTTGGACAATCCTGTCTACACTTACTTCCGTGCTTCCACTTTGAATCTTGAGACTAATGA
GTACCACCAGTACGTTACAGTGGGTTCTTCCACTCCTAATATAACTACCTTGGATGTTTCTGTGGGTAACAATG
GCATTAAAAGTGAGTCCGAAGATAATCTTTCTAAGCTGAGATCATACTCTAACCATGATAATGTGACCTTCGAT
ATTACTTACGACGCCACCACTGGAGCTGTCGCTAATGGAGGCGCCGGAACTTTCCAATTCGGAGAAGGTTTGA
CATGGGAGTTCGGTTTGCCTTCTGCTAAAACAGAGGGCTCCCTGACCGTTCATGGTGAAAAACTTGCTATCGAC
CCTGCTAAATCCCATACATGGTATGATCGTCAGTGGGGAAATACTGCCGCTATCCCCTCTAACTGGACTTGGTT
CCAACTTCATATTCCATCCACAGAATACAAAATTAGTGCATGGATTTTCTCCGACCCATTCAGAAACACAGAAAC
CCGTTTTGCAACTATTAGAGGTGCAAATGATGAAACACTGGTACTACCTTTGGAATTTACACCAATCTACAAAA
GAACCTACGAATCTGCCACCGGAAGAGTTACATATCCTCTTGATTGGAAATTGAAGATTTCCGGATTCGGTGAT
TTCAAACTTAGTTCTTATACAGAAGATCAAGAGCTAGTTGGTGAAGATGCCCTTCAAACAGCATACGAGGGAT
TCATTACCTTCAGTGGTAATGTGCACTCTAAACCCGTGCAGGGGTATGGCCTTGTGGAAATCGTTTACAGTACT
TGGGATGTCTAA

>CDS_*Fs*KHS

ATGATGATTTCATCTGTCTTGGTCGCCGGTGTTGTCGCAGTCTCTGCCGCTCTAGCTAGCAAGCATCCCAA
ACAATACTCTTTCAAGCCAGAGGATGCTGAGACCATCTGGAATGGCGACATTCCTGTATTGTACGATTTCGGTG
ACTCTCAATCTGCTTCTTACTCCGGTTCTTGGTGGACTAGTTCATACATTACTGGTACTAACGGTGAACAATATC
TAGTGATTTCTCATTATCTGGATACACCAGTTTTTACTTATTTCAGAGCATCAACTCTAAACCTGGAAACTTTGG
ATTATAATCAGTTCATTACTTTGGGTAATAATACTGCTAATTCTACTACCCTTGATGTTAAGGTCGGAGACAATG
GAATCCAAAGTCTGACCGCCGATAATATTTCTCAACAGCGTGCTTACGCAAACGATGAAAATGTCACTTTCGAT
ATCACTTTTGATGCTACATCTCGTGTTATCTCTAATGCCGGCGCTGGAGTGTTTCAGTTTGGCCCTTCTATTACCT
ATGAGTGGGGTCTTCCAAACTGTCGTACCCAGGGTTCTGTTACTGATACAGGTGGAAAAAATATCACCGTGGA
CCCTGCTAAGTCATTCACTTGGTATGACAGACAATGGGGCACAGCCGCAGTCACTTCTGGTAACTGGACTTGGT
TCCAAATGCATATTCCAGAAACTTCTTATAAGTTGAGTGTTTGGATTATCGACAACGATGTTACCAACCAGTTCT
CCCGTTTCGCCACCATCAGAGGAGACAATGATGAGTTCCAAGTGCTTCCACTGGAGTGGAAGCCAATTTATGA
CAGAACTTACCAATCTACTGCCGCTGATATCCTGTACCCATTGGATTGGGAACTTGATATCTCTGGTTTCGGAGT
CTTCCAACTTTCTTCCATTCTTGATGACCAAGAAATCGTCGGTACTACCGCCATCCAGACCGCATACGAAGGATT
TGTCACTTTTAATGGTACTGTTCATAATAAAAAGGTTCAGGGTTACGGCCTTGTGGAAGTCGTGTATTCTAATT
GGGAGTCTCTTTAA

S2 Figure. Coding sequence of codon-harmonized putative kievitone hydratases from *N. haematococca* (*Nh*KHS) and from *F. solani* (*Fs*KHS) for expression in *P. pastoris*.
