# Supplementary material for: Recombinant expression, purification and biochemical characterization of kievitone hydratase from Nectria haematococca
Source: PLoS One. 2018 Feb 8;13(2):e0192653. doi: 10.1371/journal.pone.0192653 (PMC5805349; doi:10.1371/journal.pone.0192653)
Supplement: S3 Fig — Inset: Calibration curve generated with the standard proteins conalbumin (75 kDa), ovalbumin (44 kDa), carbonic anhydrase (29 kDa), ribonuclease A (13.7 kDa) and aprotinin (6.5 kDa). (PDF) [file pone.0192653.s003.pdf]

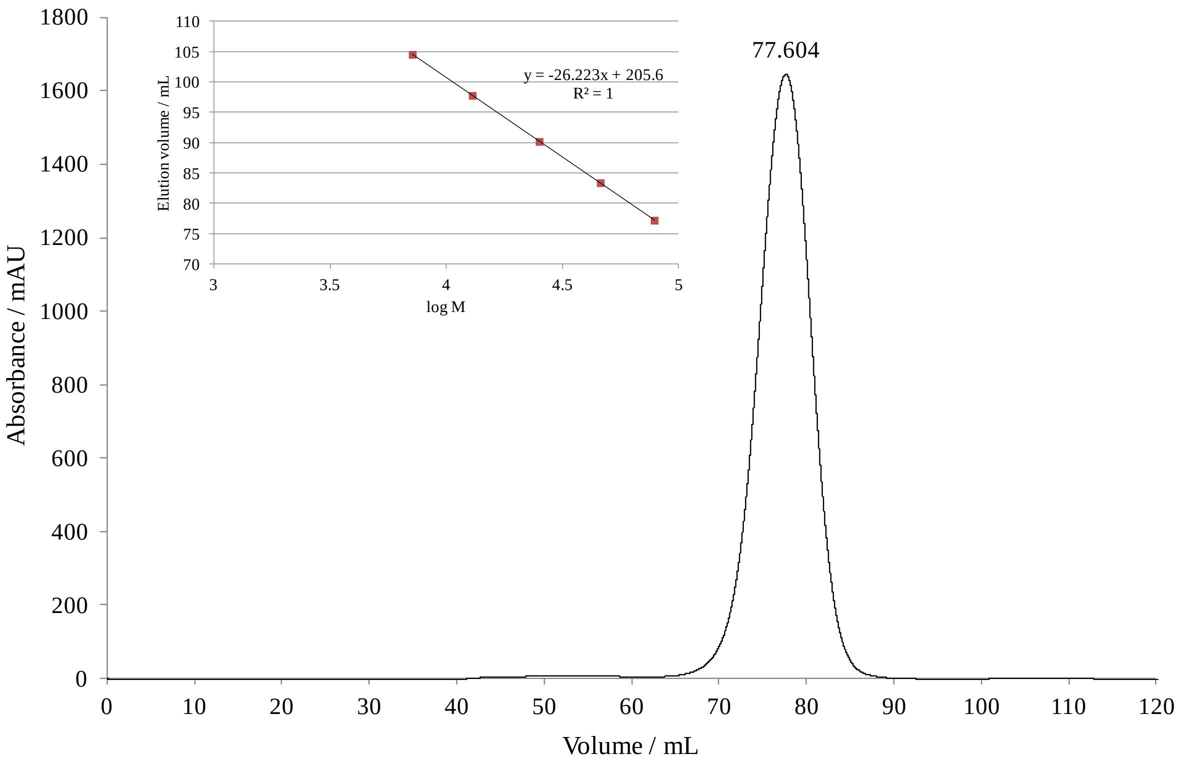

S3 Figure. Gel filtration profile of *Nh*KHS on a Superdex 200 HiLoad 16/60 column (GE Healthcare, UK) using 50 mM sodium citrate, pH 6.0, as buffer system. Inset: Calibration curve generated with the standard proteins conalbumin (75 kDa), ovalbumin (44 kDa), carbonic anhydrase (29 kDa), ribonuclease A (13.7 kDa) and aprotinin (6.5 kDa).
