# Supplementary material for: Recombinant expression, purification and biochemical characterization of kievitone hydratase from Nectria haematococca
Source: PLoS One. 2018 Feb 8;13(2):e0192653. doi: 10.1371/journal.pone.0192653 (PMC5805349; doi:10.1371/journal.pone.0192653)
Supplement: S4 Fig — After incubation of XN with purified NhKHS, the reaction products were extracted and prepared for NMR analysis as described. (A) 1H-NMR spectrum of XN at 499.8 MHz. (B) 13C-NMR spectrum of XN at 125.7 MHz. (C) 1H-NMR spectrum of HO-XN at 499.8 MHz. (D) 13C-NMR spectrum of HO-XN at 125.7 MHz. HO-XN could be unambiguously identified as the reaction product upon hydration of XN by NhKHS, and substrate and product were independently confirmed by comparison with 1H- and 13C-NMR spectra provided in other work [1,2]. (PDF) [file pone.0192653.s004.pdf]

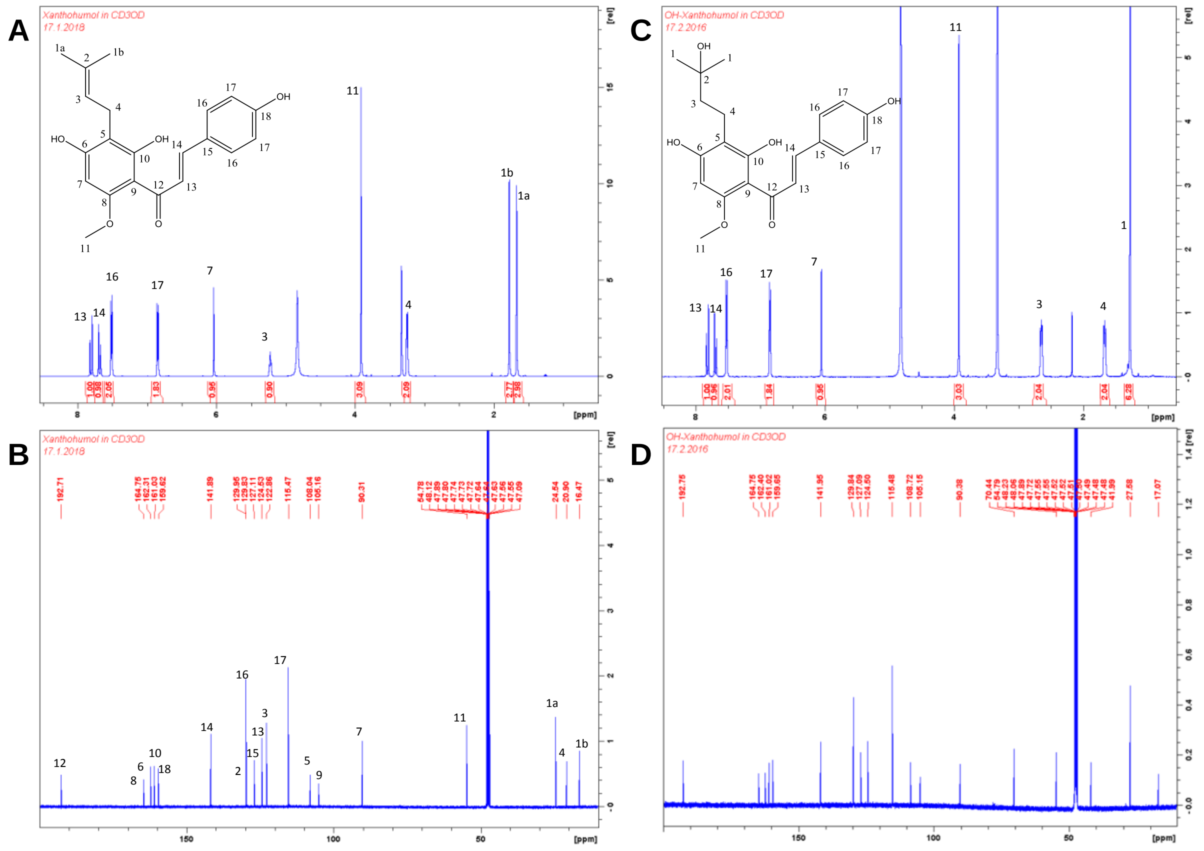

S4 Figure. ^{1}H- and ^{13}C-NMR spectra of XN and HO-XN in CD_3OD. After incubation of XN with purified *Nh*KHS, the reaction products were extracted and prepared for NMR analysis as described. (A) ^{1}H-NMR spectrum of XN at 499.8 MHz. (B) ^{13}C-NMR spectrum of XN at 125.7 MHz. (C) ^{1}H-NMR spectrum of HO-XN at 499.8 MHz. (D) ^{13}C-NMR spectrum of HO-XN at 125.7 MHz. HO-XN could be unambiguously identified as the reaction product upon hydration of XN by *Nh*KHS, and substrate and product were independently confirmed by comparison with ^{1}H- and ^{13}C-NMR spectra provided in other work.
