# Supplementary material for: Recombinant expression, purification and biochemical characterization of kievitone hydratase from Nectria haematococca
Source: PLoS One. 2018 Feb 8;13(2):e0192653. doi: 10.1371/journal.pone.0192653 (PMC5805349; doi:10.1371/journal.pone.0192653)
Supplement: S5 Fig — The standard enzyme assay was performed in the presence of respective organic solvents at concentrations ranging from 0.5 to 30%. One mM of XN and 0.05 mg mL-1 of enzyme were incubated for 3 h. Amounts of XN-hydrate in mM were obtained via HPLC-MS measurements. Biological triplicates were analyzed. (PDF) [file pone.0192653.s005.pdf]

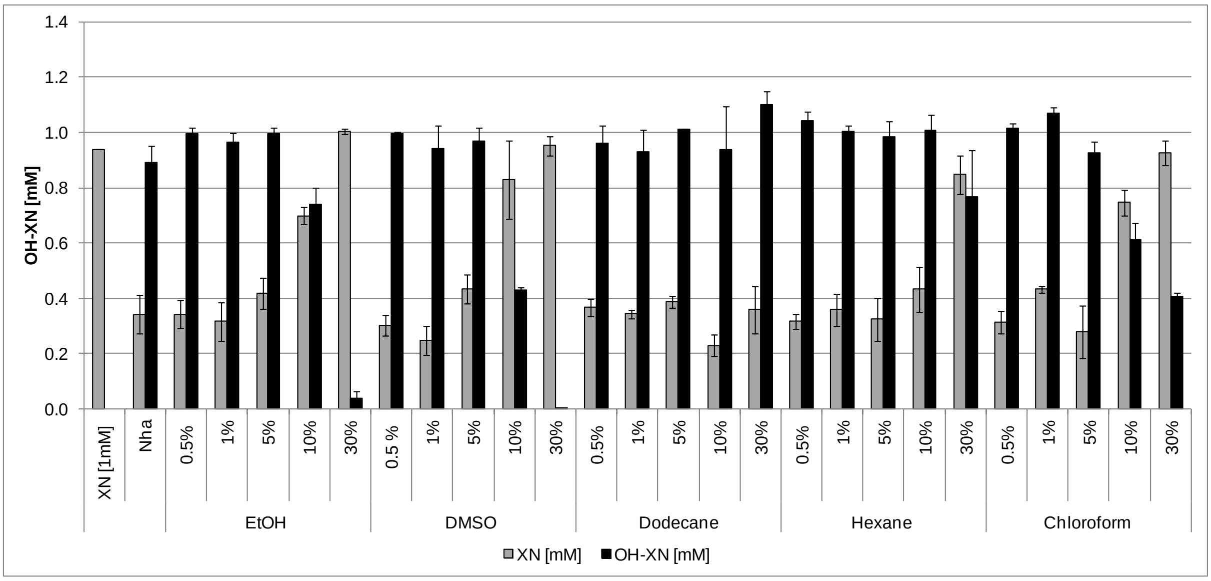

S5 Figure. Influence of different organic solvents on the activity of *Nh*KHS. The standard enzyme assay was performed in the presence of respective organic solvents at concentrations ranging from 0.5 to 30 %. One mM of XN and 0.05 mg mL^{-1} of enzyme were incubated for 3 h. Amounts of XN-hydrate in mM were obtained via HPLC-MS measurements. Biological triplicates were analyzed.
