# Supplementary material for: Recombinant expression, purification and biochemical characterization of kievitone hydratase from Nectria haematococca
Source: PLoS One. 2018 Feb 8;13(2):e0192653. doi: 10.1371/journal.pone.0192653 (PMC5805349; doi:10.1371/journal.pone.0192653)
Supplement: S6 Fig — Results of activity assays using 3 μL of supernatant of the fermentation broth from strain PpKHSAlpha at indicated time points of induction (A). Volumetric activity of strain PpKHSAlpha (B). Activity assays and HPLC analyses were performed in triplicates. NhKHS levels in culture supernatants were monitored by SDS-PAGE (C) and compared to purified protein (KHS pur.). (PDF) [file pone.0192653.s006.pdf]

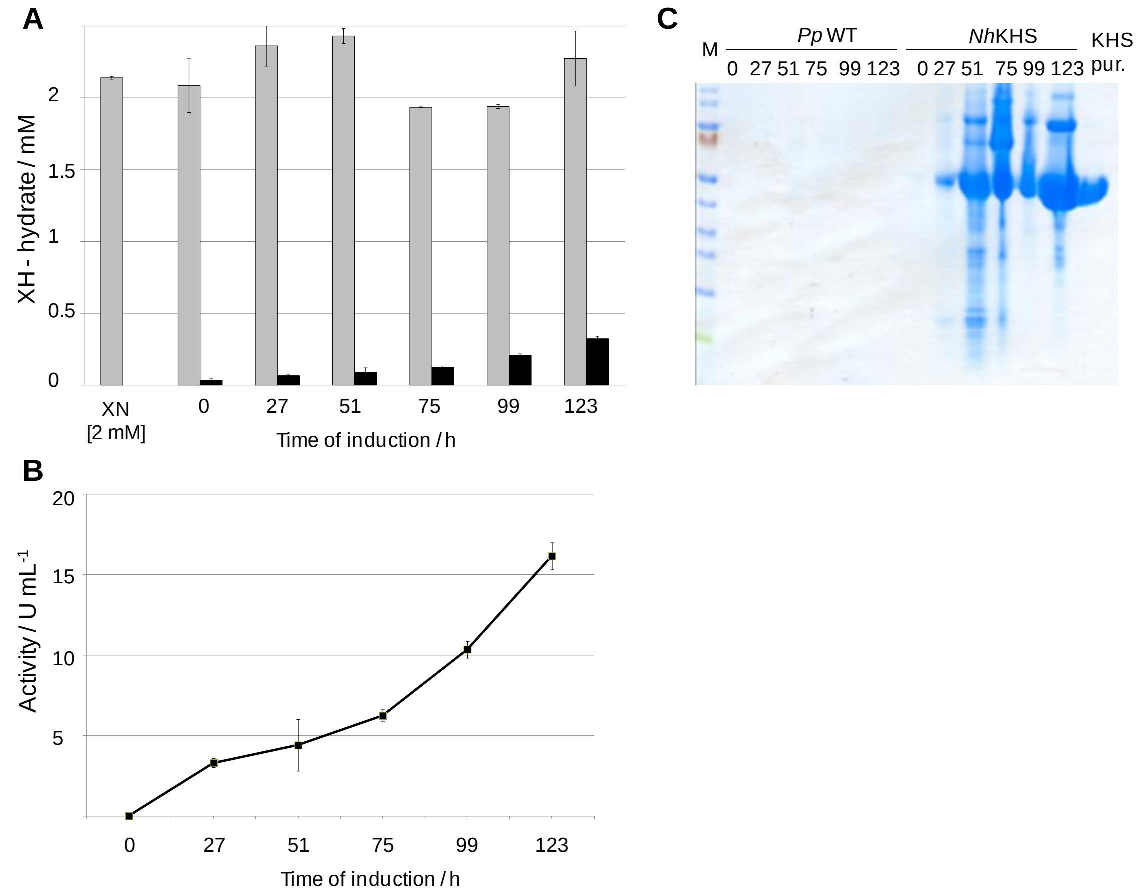

S6 Figure. Bioreactor cultivation of strain *PpKHS*Alpha. Results of activity assays using 3 µL of supernatant of the fermentation broth from strain *PpKHS*Alpha at indicated time points of induction (A). Volumetric activity of strain *PpKHS*Alpha (B). Activity assays and HPLC analyses were performed in triplicates. *Nh*KHS levels in culture supernatants were monitored by SDS-PAGE (C) and compared to purified protein (KHS pur.).
