# Supplementary material for: Recombinant expression, purification and biochemical characterization of kievitone hydratase from Nectria haematococca
Source: PLoS One. 2018 Feb 8;13(2):e0192653. doi: 10.1371/journal.pone.0192653 (PMC5805349; doi:10.1371/journal.pone.0192653)
Supplement: S1 Table — (PDF) [file pone.0192653.s007.pdf]

S1 Table. Primers used for cloning of *Nh*KHS and *FsKHS* into the vector p*Pp*T4_Alpha_S.

Primer name	Primer (5‘ – 3‘)
Fw(alpha_XhoI_*Nh*KHS)	TCTCTCGAGAAGAGAGAGGCCGAAGCTATGAGAGCTTCCTTTCTTCTG
Fw(alpha_XhoI_*Fs*KHS	TCTCTCGAGAAGAGAGAGGCCGAAGCTATGATGATTTCATCTGTCTTGG
Rv(NotI_Pp_HisKHS)	GAGCGGCCGCCCTTTAGTGATGGTGATGGTGATG
