# Supplementary material for: Recombinant expression, purification and biochemical characterization of kievitone hydratase from Nectria haematococca
Source: PLoS One. 2018 Feb 8;13(2):e0192653. doi: 10.1371/journal.pone.0192653 (PMC5805349; doi:10.1371/journal.pone.0192653)
Supplement: S2 Table — Purified enzyme was deglycosylated with EndoHf enzyme mix for 0.5 h at 37°C. (PDF) [file pone.0192653.s008.pdf]

S2 Table. Thermostability of glycosylated and deglycosylated *Nh*KHS at different pH values. Purified enzyme was deglycosylated with EndoH_f enzyme mix for 0.5 h at 37°C.

pH	T_m (°C)	
	***Nh*KHS**	***Nh*KHS - deglycosylated**
4.0	15.7 ± 1.2	15.0 ± 0.0
5.0	49.3 ± 0.6	49.0 ± 0.0
6.0	50.3 ± 0.6	49.0 ± 0.0
7.0	44.3 ± 2.1	43.3 ± 2.1
8.0	15 ± 0.0	15.0 ± 0.0
9.0	11.3 ± 0.6	12.3 ± 2.3
